# Anticancer and Antioxidant Activities of the Root Extract of the Carnivorous Pitcher Plant *Sarracenia purpurea*

**DOI:** 10.3390/plants11131668

**Published:** 2022-06-23

**Authors:** Yen-Hua Huang, Wei-Yu Chiang, Pin-Jui Chen, En-Shyh Lin, Cheng-Yang Huang

**Affiliations:** 1 Department of Biomedical Sciences, Chung Shan Medical University, Taichung City 402, Taiwan; cicilovev6@gmail.com (Y.-H.H.); www333444777@gmail.com (W.-Y.C.); sean742742@gmail.com (P.-J.C.); 2 Department of Beauty Science, National Taichung University of Science and Technology, Taichung City 403, Taiwan; eslin7620@gmail.com; 3 Department of Medical Research, Chung Shan Medical University Hospital, Taichung City 402, Taiwan

**Keywords:** carnivorous plant, *Sarracenia purpurea*, anticancer, 4T1 mammary carcinoma, antioxidation, antibacterial, dihydroorotase, SSB, GC–MS analysis, ionone

## Abstract

The carnivorous pitcher plant *Sarracenia purpurea* exhibits many ethnobotanical uses, including the treatments of type 2 diabetes and tuberculosis-like symptoms. In this study, we prepared different extracts from the leaves (pitchers), stems, and roots of *S. purpurea* and investigated their antioxidant and anticancer properties. To evaluate the extraction efficiency, we individually used different solvents, namely methanol, ethanol, acetone, and distilled water, for *S. purpurea* extract preparations. The root extract of *S. purpurea*, obtained by 100% acetone (*S. purpurea-root-acetone*), had the highest anticancer activities, antioxidation capacity (the DPPH activity with IC_50_ of 89.3 ± 2.2 μg/mL), antibacterial activities, total phenolic content (33.4 ± 0.7 mg GAE/g), and total flavonoid content (107.9 ± 2.2 mg QUE/g). The most abundant compounds in *S. purpurea-root-acetone* were identified using gas chromatography–mass spectrometry; 7,8-Dihydro-α-ionone was the major compound present in *S. purpurea-root-acetone*. In addition, the co-cytotoxicity of *S. purpurea-root-acetone* (combined with the clinical anticancer drug 5-fluorouracil (5-FU) on the survival, apoptosis, proliferation, and migration of the 4T1 mammary carcinoma) was examined. The combination of 5-FU with *S. purpurea-root-acetone* could be highly efficient for anti-4T1 cells. We also found that *S. purpurea-root-acetone* could inhibit the enzymatic activity of human dihydroorotase (huDHOase), an attractive target for potential anticancer chemotherapy. The sic most abundant compounds in *S. purpurea-root-acetone* were tested using an in silico analysis via MOE-Dock software for their binding affinities. The top-ranked docking conformations were observed for 7,8-dihydro-α-ionone and stigmast-5-en-3-ol, suggesting the inhibition potential against huDHOase. Overall, the collective data in this study may indicate the pharmacological potentials of *S. purpurea-root-acetone* for possible medical applications.

## 1. Introduction

**Figure 1:**
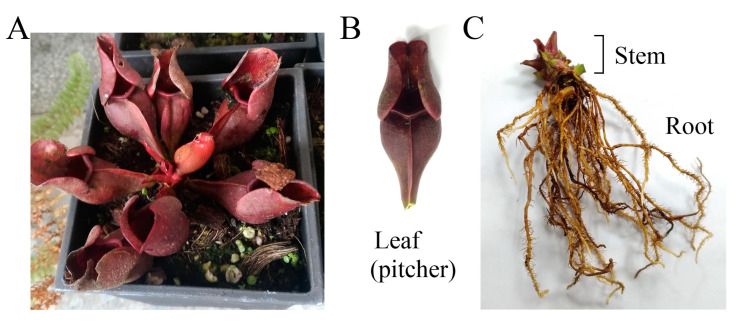
Preparation of different extracts from *Sarracenia purpurea*. (**A**) *S. purpurea*. It is a carnivorous pitcher plant with cone-shaped leaves for obtaining supplemental nutrients. (**B**) Leaf of *S. purpurea*. (**C**) Stem and root. This plant was photographed at a magnification of 0.48. Different parts of *S. purpurea*, including the leaves, stems, and roots, were collected, dried, cut into small pieces, and pulverized into powder. Extractions were carried out by using methanol, ethanol, acetone, and distilled water.

*Sarracenia purpurea* is a carnivorous pitcher plant with cone-shaped leaves used for obtaining supplemental nutrients [1]. To adapt to nutrient-poor habitats, *S. purpurea* attracts, traps, and digests prey, to absorb their soluble ingredients for growth and reproduction. The leaf extract of *S. purpurea* exhibits several ethnobotanical uses in many aboriginal communities. For example, the leaf extract of *S. purpurea* is a traditional medicine for the treatment of type 2 diabetes [2]. The leaf extract of *S. purpurea* obtained using methanol also possesses antimycobacterial activity for the treatment of tuberculosis-like symptoms [3]. Given that many kinds of natural extracts from plants have antimicrobial activities [4,5,6,7,8], it is worth determining the antibacterial properties of *S. purpurea*. Prior to this study, whether leaves, stems, or roots of *S. purpurea* (Figure 1) had broad ranges of antibacterial activities against human pathogens remained uninvestigated, and should be elucidated.

Cancer is known as one of the most life-threatening diseases worldwide. Cancer mortality is on the rise and has become one of the leading causes of human mortality [9,10]. Although substantial progress has been made in the control and treatment of cancer, it still caused approximately 9.9 million deaths in 2020. Conventional cancer treatments commonly involve chemotherapy or a combination of radiotherapy and chemotherapy. Several adverse effects associated with modern chemotherapy hinder cancer treatment and lead to other critical disorders. Therefore, natural compounds as potential anticancer agents are also used and studied in many cancer models, both in vitro and in vivo [11,12,13,14]. Plant-based medicines have been employed in clinical practices and have yielded good results without causing significant adverse effects [15]. Phytochemicals, such as vincristine, vinblastine, paclitaxel, curcumin, colchicine, and lycopene, have shown promising anticancer effects [15,16]. Recent findings indicated that extracts of the carnivorous pitcher plant *Nepenthes miranda* exhibited cytotoxicity on cancer cell survival, migration, proliferation, and induced apoptosis [17,18]. Given that *S. purpurea* is also a pitcher plant, it is worth determining the anticancer properties of *S. purpurea*. Currently, it is unknown whether extracts of *S. purpurea* have anticancer activities.

Phytochemicals, which are present in many herbs, have received attention due to their health benefits [19]. One significant advantage of using natural extracts against cancer cells is their multitargeted modes of action, which provide potential synergistic behavior and polypharmacology approaches for cancer therapies. Many natural compounds exhibiting anticancer properties are phenols that can influence cell cycles. Phenolic compounds are the main class of secondary metabolites in plants and are divided into phenolic acids and polyphenols [20,21,22]. Flavonoids are a family of polyphenolic compounds, many of which display structure-dependent biological and pharmacological activities [23,24]. The antioxidant activities of polyphenols, due to their abilities to scavenge free radicals, donate hydrogen atoms or electrons, or chelate metal cations, are key factors in combating cellular oxidative stress. Thus, the total phenolic content (TPC), total flavonoid content (TFC), and antioxidation activities of different extracts were also determined in this study to evaluate the pharmacological potentials of *S. purpurea* for possible medical applications. The root extract of *S. purpurea* obtained by 100% acetone—owing to the highest anticancer activities, TPC, TFC, and antioxidation capacity—was chosen for GC–MS analysis to determine the presence of medicinally active ingredients; we found that 7,8-dihydro-α-ionone, 24-norursa-3,12-diene, α-amyrin, stigmast-5-en-3-ol, botulin, and 24-noroleana-3,12-diene were the most abundant compounds (>1.4%) in this extract.

## 2. Results

*S. purpurea* is employed as a traditional medicine to treat symptoms of diabetes in many aboriginal communities [25]. Prior to this study, anticancer and antioxidant activities of the extract of this carnivorous plant were not investigated. The current study aimed to uncover cytotoxicity in the root extract from *S. purpurea*. The cytotoxicity activities of the root extract combined with the clinical anticancer drug 5-fluorouracil (5-FU) against cancer cells were also studied.

### 2.1. Total Phenolic Content (TPC)

Many polyphenols can be developed as drug candidates from the active confirmation of in vitro screens or in vivo evaluations [26]. The total phenolic content (TPC) of *S. purpurea* remained undetermined and, thus, was analyzed by using different *S. purpurea* extracts. TPC was quantified using the modified Folin–Ciocalteu method. Various parts of *S. purpurea* (leaves, stems, and roots) were extracted using different solvents (water, methanol, ethanol, and acetone) and analyzed for their TPC. For clarity, the extract was designated as *S. purpurea*-which part-which solvent used. TPC values ranged from 3.2 mg GAE/g for *S. purpurea-root-water* to 33.4 mg GAE/g for *S. purpurea-root-acetone* (Table 1). Thus, the root was the major source of phenols in *S. purpurea*.

### 2.2. Total Flavonoid Content (TFC)

Many flavonoids display structure-dependent biological and pharmacological activities [24]. Thus, the total flavonoid content (TFC) of *S. purpurea* was also determined (Table 2). TFC was quantified using the aluminum chloride colorimetric method. TFC values ranged from 5.6 mg QUE/g for *S. purpurea-root-water* to 107.9 mg QUE/g for *S. purpurea-root-acetone*. Overall, the root was the major source of phenols (Table 1) and flavonoids (Table 2) in *S. purpurea*.

### 2.3. Antioxidant Activity

**Figure 2:**
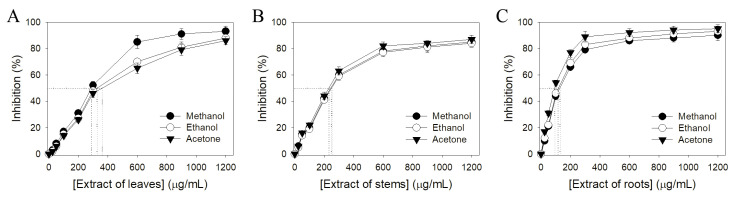
Antioxidant activity of *S. purpurea* extracts. The antioxidant activities of extract from (**A**) leaves, (**B**) stems, and (**C**) roots were evaluated by DPPH radical scavenging assay.

The antioxidant activities of different *S. purpurea* extracts were evaluated by the DPPH radical scavenging assay (Figure 2). DPPH assay is the most common method to assess the antioxidant capacity of plants. The antioxidant properties of different *S. purpurea* extracts were described by IC_50_ values; the lower IC_50_ value indicated the higher radical scavenging activity (Table 3). IC_50_ values were calculated from the titration curves of the DPPH assay by determining the concentration of the extract needed to achieve the midpoint value for inhibition (Figure 2). Due to <30% inhibition at a concentration of 600 μg/mL, we did not determine the IC_50_ values of water extracts of *S. purpurea*. According to their IC_50_ values (Table 3), the antioxidant capacity of *S. purpurea* extracts followed the order: roots > stems > leaves. *S. purpurea-root-acetone* showed the highest antioxidant capacity with an IC_50_ value of 89.3 ± 2.2 μg/mL.

### 2.4. Antibacterial Activity

The antibacterial activities of different *S. purpurea* extracts were investigated using the agar well diffusion method. Human pathogens (*Staphylococcus aureus*, *Pseudomonas aeruginosa*, and *Escherichia coli*) were used for this analysis. The extracts showed differences in antibacterial activities with the zone of inhibition ranging from 7 to 15 mm (Table 4). The water extracts of *S. purpurea* did not the inhibit growth of these bacteria. Extracts of *S. purpurea* leaves did not inhibit the growth of *S. aureus*. *S. purpurea-root-acetone* showed the highest antibacterial activities against these three bacteria.

### 2.5. Anticancer Potential

**Figure 3:**
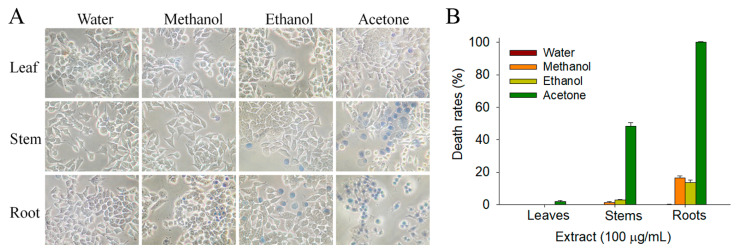
The cytotoxic effects of different *S. purpurea* extracts against 4T1 cells. (**A**) Trypan blue dye exclusion staining. The cytotoxic effects of different *S. purpurea* extracts against 4T1 cells were estimated with trypan blue assay after 24 h of incubation. The 4T1 cells incubated with *S. purpurea-root-acetone* of 100 mg/mL were almost dead. (**B**) The death rates of 4T1 cells. The anti-4T1 activity of *S. purpurea* extracts followed the order: roots > stems > leaves. The solvent used for extract preparations was also an important factor. As compared, the acetone fractions showed the highest anti-4T1 activities. The water extracts of *S. purpurea* did not cause any cytotoxic effects on the survival of 4T1 cells.

Prior to this study, whether extracts of *S. purpurea* could suppress the growth of cancer cells was undetermined. The 4T1 cells were used for this investigation. The 4T1 mammary carcinoma is a transplantable breast cancer cell line that is highly tumorigenic and invasive [27,28]. In contrast to most tumor models, 4T1 cells can spontaneously metastasize from the primary tumor in the mammary gland to multiple distant sites, including the lymph nodes, blood, liver, lung, brain, and bone [29,30]. Different *S. purpurea* extracts were used to determine the cytotoxic effects against 4T1 cells (Figure 3). The monolayers prepared in 96-well microtitration plates for 4T1 cells were inoculated with different *S. purpurea* extracts at concentrations of 100 μg/mL per well. The cytotoxic effects of *S. purpurea* extracts were estimated with trypan blue staining assay after 0 and 24 h of incubation (Figure 3A). The anti-4T1 activity of *S. purpurea* extracts followed the order: roots > stems > leaves. The solvent used for extract preparations was also an important factor. As compared, the acetone fractions showed the highest anti-4T1 activities (Figure 3B). The water extracts of *S. purpurea* did not cause any cytotoxic effect on the survival of 4T1 cells.

### 2.6. Apoptosis of 4T1 Cells Induced by S. purpurea-root-acetone

**Figure 4:**
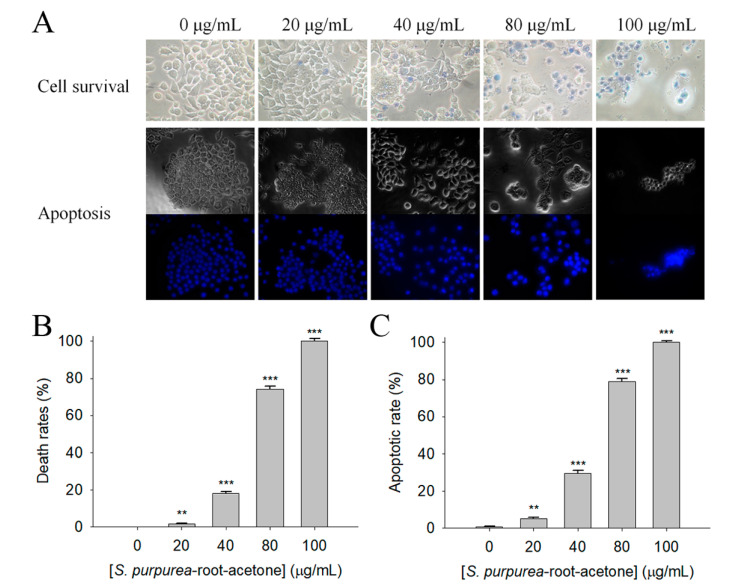
The cytotoxic effect of *S. purpurea-root-acetone* against 4T1 cells. (**A**) Effects of different concentrations of *S. purpurea-root-acetone* on cell survival and nuclear condensation. (**B**) Trypan blue dye exclusion staining; 4T1 cells incubated with *S. purpurea-root-acetone* at different concentrations. (**C**) Hoechst staining. Different concentrations of *S. purpurea-root-acetone*-induced apoptosis with DNA fragmentation were observed in 4T1 cells. ** *p* < 0.01 and *** *p* < 0.001 compared with the control group.

The initial screening using different *S. purpurea* extracts for possible anticancer activity through the trypan blue assay showed that 100 μg/mL of *S. purpurea-root-acetone* could cause significant deaths of 4T1 cells (Figure 3). Different concentrations of *S. purpurea-root-acetone* (0, 20, 40, 80, and 100 μg/mL) were used to further investigate the cytotoxic effects (Figure 4A). Incubation with *S. purpurea-root-acetone* of 0, 20, 40, 80, and 100 μg/mL caused deaths of 4T1 cells by 0, 2, 18, 74, and 100%, respectively (Figure 4B). Based on these results, the 50% lethal concentration (LC_50_) of *S. purpurea-root-acetone* was estimated to be 63 ± 2 μg/mL. *S. purpurea-root-acetone*-induced nuclear condensation, a process to distinguish apoptotic cells, was also examined. We found that incubation with *S. purpurea-root-acetone* can induce apoptosis of 4T1 cells. Through the Hoechst staining assay, we found that 80 and 100 μg/mL of *S. purpurea-root-acetone* could induce apoptosis (Figure 4C) with DNA fragmentation in 4T1 cells by 79 and 100%, respectively.

### 2.7. Apoptosis of Melanoma Cancer Cells Induced by S. purpurea-root-acetone

**Figure 5:**
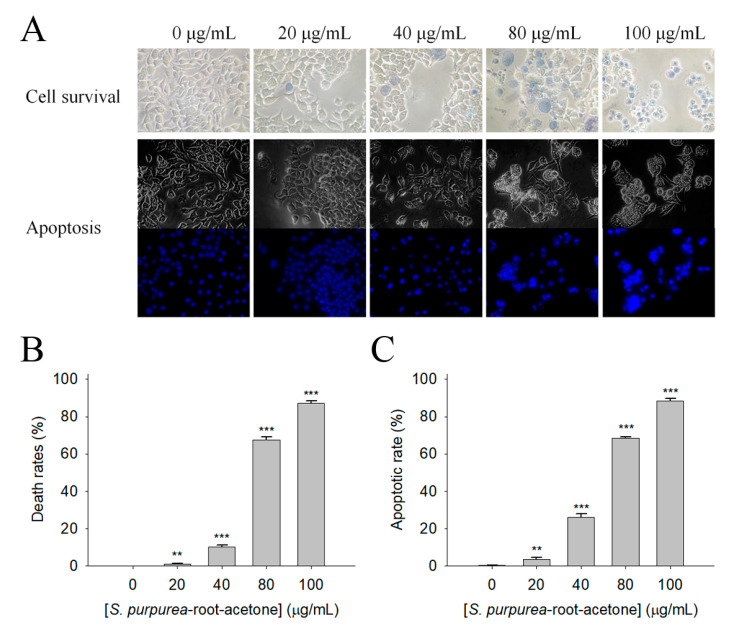
The cytotoxic effect of *S. purpurea-root-acetone* against B16F10 cells. (**A**) Effects of different concentrations of *S. purpurea-root-acetone* on cell survival and nuclear condensation. (**B**) Trypan blue dye exclusion staining. B16F10 cells incubated with *S. purpurea-root-acetone* at different concentrations. (**C**) Hoechst staining. Different concentrations of *S. purpurea-root-acetone*-induced apoptosis with DNA fragmentation were observed in B16F10 cells. ** *p* < 0.01 and *** *p* < 0.001 compared with the control group.

Malignant melanoma is the most dangerous (and the most common) type of skin cancer [31,32]. Melanoma B16F10 cells are highly metastatic. Considering that many natural products exhibit anticancer properties for skin cancers [32], whether *S. purpurea-root-acetone* could inhibit the growth of melanoma cells (Figure 5A) was also investigated in this study. The trypan blue assay showed that incubation with *S. purpurea-root-acetone* at 0, 20, 40, 80, and 100 μg/mL caused the deaths of B16F10 cells by 0, 1, 11, 67, and 87%, respectively (Figure 5B). The LC_50_ of *S. purpurea-root-acetone* was estimated to be 68 ± 3 μg/mL. Incubation with *S. purpurea-root-acetone* also induced apoptosis of B16F10 cells. Through the Hoechst staining assay, we found that 80 and 100 μg/mL of *S. purpurea-root-acetone* could induce apoptosis (Figure 5C) with DNA fragmentation in B16F10 cells by 69 and 88%, respectively.

### 2.8. Potential Synergistic Anticancer Effects of S. purpurea-root-acetone with 5-FU

**Figure 6:**
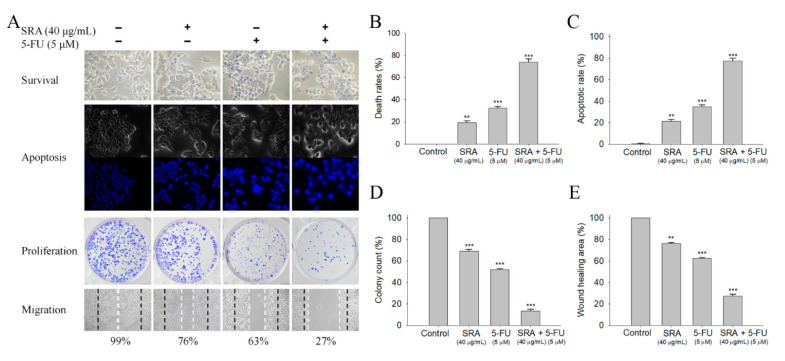
The synergistic anticancer effects of *S. purpurea-root-acetone* and 5-FU. (**A**) Effects of *S. purpurea-root-acetone* and 5-FU on cell survival, migration, proliferation, and apoptosis. (**B**) Trypan blue dye exclusion staining; 4T1 cancer cells incubated with *S. purpurea-root-acetone* and 5-FU. (**C**) Hoechst staining. *S. purpurea-root-acetone*- and 5-FU-induced apoptosis with DNA fragmentation was observed in 4T1 cells. (**D**) Clonogenic formation assay. Pretreatment with *S. purpurea-root-acetone* and 5-FU significantly suppressed the proliferation and colony formation of 4T1 cells. (**E**) The wound-healing assay. *S. purpurea-root-acetone* and 5-FU significantly inhibited cell migration. ** *p* < 0.01 and *** *p* < 0.001 compared with the control group.

The FDA-approved clinical drug 5-FU has remarkable therapeutic effects against different cancers [33]. In addition, the combination of 5-FU with natural products, i.e., myricetin [34], sinomenine [35], lapatinib [36], or curcumin [37] could be highly efficient for cancer therapies [38]. We investigated whether *S. purpurea-root-acetone* could act with 5-FU against 4T1 cancer cells (Figure 6A). The co-cytotoxic effects of *S. purpurea-root-acetone* with 5-FU on survival (Figure 6B), apoptosis (Figure 6C), proliferation (Figure 6D), and migration (Figure 6E) of 4T1 cells were examined. The migration and proliferation of 4T1 cells were analyzed by clonogenic formation and wound-healing assays, respectively. *S. purpurea-root-acetone* (40 μg/mL) alone could cause 4T1 cell deaths by 19%, induce apoptosis by 21%, suppress proliferation by 31% (69% colony formation), and inhibit cell migration by 24% (76% migration), respectively. We found that 5-FU acting with *S. purpurea-root-acetone* had synergistic anti-4T1 cancer cell effects. The cytotoxic effect was significantly enhanced (74% cell mortality) when *S. purpurea-root-acetone* (40 μg/mL) was combined with 5-FU (5 μM). The cotreatment of *S. purpurea-root-acetone* with 5-FU on 4T1 cells also had greater effects on the apoptotic rate (77%), colony formation (13%), and cell migration (27%). Thus, the usage of *S. purpurea-root-acetone* with 5-FU resulted in increased cytotoxicity against 4T1 cells.

### 2.9. GC–MS Analysis of S. purpurea-root-acetone

*S. purpurea-root-acetone*—owing to its highest anticancer activities, TPC, TFC, and antioxidation capacity—was chosen for the GC–MS analysis to determine the presence of medicinally active ingredients. In the GC–MS analysis, 7,8-dihydro-α-ionone (84.4%), 24-norursa-3,12-diene (5.0%), α-amyrin (3.6%), stigmast-5-en-3-ol (3.1%), botulin (2.4%), and 24-noroleana-3,12-diene (1.4%) were the most abundant compounds (>1.4%) in *S. purpurea-root-acetone* (Table 5). Moreover, 7,8-dihydro-α-ionone (84.4%) was the major compound present in *S. purpurea-root-acetone*.

### 2.10. Dihydroorotase Inhibitory Potential

Dihydroorotase (DHOase) [17,39,40,41] is the third enzyme in the de novo biosynthesis pathway for pyrimidine nucleotides [42] and an attractive target for potential anticancer chemotherapy [43,44]. When the urea cycle is dysregulated, nitrogen will be redirected and used by the multifunctional enzyme CAD (carbamoyl phosphate synthetase/aspartate transcarbamoylase/DHOase) to increase pyrimidine synthesis in cancer cells [45]. Thus, we tested whether the enzymatic activity of the DHOase domain of human CAD (huDHOase) can be inhibited by any extract of *S. purpurea*. By using the standard assay, *S. purpurea-root-acetone* showed huDHOase inhibition by approximately 30% at a 30 μg/mL concentration, whereas other extracts exhibited negligible inhibitions. This result might indicate that certain compound(s) in *S. purpurea-root-acetone* can be potential huDHOase inhibitors.

In GC–MS analysis, 7,8-dihydro-α-ionone, 24-norursa-3,12-diene, α-amyrin, stigmast-5-en-3-ol, betulin, and 24-noroleana-3,12-diene were the most abundant compounds (>1.4%) in *S. purpurea-root-acetone* (Table 5). It was tentatively speculated that one or more of these compounds is responsible for this anti-huDHOase activity. We then analyzed their binding capacity via the MOE-Dock tool in a molecular operating environment (MOE) [46]. In the MOE, receptor–ligand binding affinities with all possible binding geometries are predicted on the basis of the docking score (the S score). As shown in Table 6, the S scores for 7,8-dihydro-α-ionone, 24-norursa-3,12-diene, α-amyrin, stigmast-5-en-3-ol, betulin, and 24-noroleana-3,12-diene were −6.3427, −5.6540, −6.0751, −6.8351, −5.8655, and −5.8697, respectively. Based on the docking results, the binding capacity of these compounds followed the order: stigmast-5-en-3-ol > 7,8-dihydro-α-ionone > α-amyrin > 24-noroleana-3,12-diene > betulin > 24-norursa-3,12-diene (Table 6). Thus, stigmast-5-en-3-ol might exhibited the greatest binding affinity to huDHOase among these selected compounds. However, this speculation needs to be confirmed by further biochemical and structural experiments.

## 3. Discussion

The purple carnivorous pitcher plant *S. purpurea* (Figure 1) is a medicinal plant used by the Canadian First Nations to treat a wide variety of illnesses. The people of eastern Canada have traditionally used infusions of *S. purpurea* for the treatment of tuberculosis-like symptoms. [3]. The leaf extracts of *S. purpurea* are also used for the treatment of type 2 diabetes [2]. However, few studies are available on other pharmacological applications of *S. purpurea*. The extracts of *S. purpurea*, due to their (long-time) ethnomedicinal uses, are safe as pharmaceuticals and should have fewer side effects for human use. In this study, we analyzed the antioxidant activities, TFC, TPC, antibacterial and anticancer potentials, and anti-DHOase activities of different extracts of *S. purpurea*. Given the need for insect attraction and contact, *S. purpurea* may evolve to have cytotoxicity to suppress any contamination by unwanted microbes from insects. Similar to the carnivorous pitcher plant *N. miranda* [17,18], *S. purpurea* also exhibited cytotoxicity on cancer cell survival, migration, and proliferation (Figure 6). The preliminary data in this study indicate that *S. purpurea-root-acetone* could be a potential natural alternative or a complementary therapy for mammary carcinoma (Figure 4) and melanoma cancers (Figure 5). The active component(s) in *S. purpurea* should be isolated and identified for further pharmacological applications.

TPC values ranged from 3.2 mg GAE/g for *S. purpurea-root-water* to 33.4 mg GAE/g for *S. purpurea-root-acetone*. TFC values ranged from 5.6 mg QUE/g for *S. purpurea-root-water* to 107.9 mg QUE/g for *S. purpurea-root-acetone*. Accordingly, the root was the major source of phenols (Table 1) and flavonoids (Table 2) in *S. purpurea*. However, we found that the solvent used for extract preparations was also an important factor. For example, the scenario for TPC (among the different plant parts studied using ethanol as solvent) was in the following order: stems (14.7 mg GAE/g) > leaves (12.5 mg GAE/g) > roots (12.1 mg GAE/g), i.e., roots were found to be poor sources of phenols. Accordingly, acetone is a useful extraction solvent for higher TPC (Table 1) and TFC (Table 2) of *S. purpurea*.

The extract of *S. purpurea-root-acetone* showed the highest DPPH radical scavenging activity with an IC_50_ value of 89.3 μg/mL, while the leaf extracts had poor radical scavenging potential (Table 3). *S. purpurea-root-acetone* was chosen for the GC–MS analysis to determine the active ingredients (Table 5), owing to its highest TPC, TFC, antioxidant capacity, and antibacterial activity (Table 4). The top content in *S. purpurea-root-acetone* was 7,8-dihydro-α-ionone (84.43%). Moreover, 7,8-dihydro-α-ionone is also abundant in some plants, such as *Persicaria hydropiper* [47] and *Ecballium elaterium* [48]. Furthermore, 7,8-dihydro-α-ionone is a phenolic compound and known to contribute to the plant’s antioxidant effects [48,49]. Other abundant compounds (>2.4%) in *S. purpurea-root-acetone* (Table 5), i.e., 24-norursa-3,12-diene [50], α-amyrin [51], stigmast-5-en-3-ol [52], botulin [53], and 24-noroleana-3,12-diene also possess antioxidant activities. Therefore, the antioxidation capacity of *S. purpurea-root-acetone* might be from the co-effects of these compounds.

**Figure 7:**
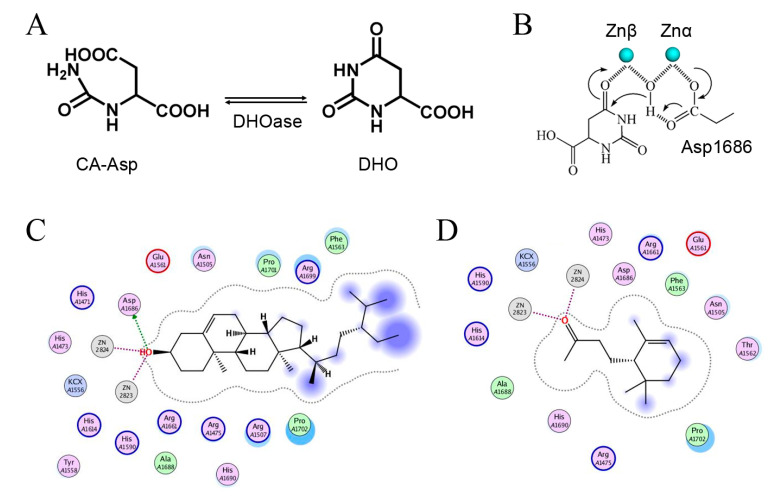
Inhibition of dihydroorotase by *S. purpurea-root-acetone*. (**A**) DHOase catalyzes the reversible cyclization of *N*-carbamoyl aspartate (CA-asp) to dihydroorotate (DHO), for the biosynthesis of pyrimidine nucleotides. (**B**) The reaction mechanism of DHOase. This enzyme contains a binuclear metal center (Znα/Znβ) and a residue Asp1686 crucial for catalysis. The hydrolysis of DHO undergoes three steps: the hydrolytic water molecule must be activated for the nucleophilic attack, the amide bond of the substrate must be made more electrophilic by polarization of the carbonyl oxygen bond, and the leaving-group nitrogen must be protonated as the carbon–nitrogen bond is cleaved. (**C**) Docking of stigmast-5-en-3-ol in the active site of huDHOase via the MOE-Dock tool. The docking model showed that the binding of stigmast-5-en-3-ol involved Znα, Znβ, and Asp1686 within the active site of huDHOase. (**D**) Docking of 7,8-dihydro-α-ionone in the active site of huDHOase via the MOE-Dock tool. The docking model showed the binding of 7,8-dihydro-α-ionone involved Znα and Znβ within the active site of huDHOase.

In this study, we identified that *S. purpurea-root-acetone* could inhibit the enzymatic activity of huDHOase. DHOase is the third enzyme in the de novo biosynthesis pathway of pyrimidine nucleotides (Figure 7A) and is considered an attractive target for potential antimalarial, anticancer, and antipathogen chemotherapy [17,39,40,41,43,54,55,56,57,58,59,60,61]. This enzyme contains a binuclear metal center (Znα/Znβ) and a residue Asp1686 (Figure 7B) crucial for the catalysis [42,62,63,64,65]. Asp1686 initiates the reaction by abstracting a proton from the substrate [66,67]. Znα and Znβ are essential for substrate-binding and hydrolysis by DHOase. Thus, the docking model with the highest score (Table 6) showed that the inhibition of huDHOase by stigmast-5-en-3-ol involved Znα, Znβ, and Asp1686 was reasonable (Figure 7C and Table 6). Perhaps the binding of stigmast-5-en-3-ol to Znα, Znβ, and Asp1686 within the active site of huDHOase would influence the substrate recognition. Inhibition of huDHOase by 7,8-dihydro-α-ionone, possibly involved in binding Znα and Znβ (Figure 7D and Table 6), was also reasonable. Further research can directly focus on determining whether stigmast-5-en-3-ol and 7,8-dihydro-α-ionone could inhibit DHOases.

We also found *S. purpurea-root-acetone* capable of inhibiting the DNA-binding activity of the single-stranded DNA-binding proteins (SSB) (unpublished results). SSB is essential for DNA replication and cell survival and, thus, is an attractive target for potential antipathogen chemotherapy [68,69,70,71]. Previously, we identified that SSB could be inhibited by the natural products myricetin [68,71] and taxifolin [69]. We will further demonstrate which compounds in *S. purpurea-root-acetone* can inhibit the DNA-binding activity of SSB.

Many phenolic compounds and metabolites occurring naturally in plants can be effective for humans in treating various disorders due to their antioxidant, anti-inflammatory, antibacterial, and anticancer activities. We found the cytotoxicity of *S. purpurea-root-acetone* against B16F10 and 4T1 cancer cells. By analyzing GC–MS, 7,8-dihydro-α-ionone (Table 5) was the major compound present in *S. purpurea-root-acetone*. Moreover, 7,8-dihydro-α-ionone is a strong antioxidant [48,49]. Recent studies have demonstrated anti-proliferative-, anti-metastatic-, and apoptosis-induction properties of β-ionone, in vitro and in vivo [72]. However, whether 7,8-dihydro-α-ionone has anticancer activity remains unknown. It will be worth determining the potential anticancer activity of 7,8-dihydro-α-ionone.

Betulin in *S. purpurea-root-acetone* is an abundant, naturally-occurring triterpene that exhibits cytotoxicity against several tumor cell lines by inducing apoptosis in cells [73,74]. Apoptotic and antiproliferative activities of stigmast-5-en-3-ol against human breast cancer MCF-7 cells are well established [75]. Accordingly, *S. purpurea-root-acetone* might induce apoptosis and cause cancer cell deaths via the co-activities of betulin and stigmast-5-en-3-ol.

Previously, we have solved complexed crystal structures of DHOase [41], SSB [76], and dihydropyrimidinase [77] with 5-FU, and structurally extended the interactome of 5-FU. Moreover, 5-FU was universally used as an anticancer agent [33,38,78]; 5-FU is a potent antimetabolite that causes RNA miscoding and inhibits DNA synthesis [79], which is also involved in increasing the intracellular reactive oxygen species (ROS)-related radical anion O_2_ level [33,80]. ROS can induce apoptotic cell death via a p53-dependent pathway [81,82]. In this study, we found that the combination of *S. purpurea-root-acetone* with 5-FU could synergistically enhance the cytotoxicity against 4T1 cells (Figure 6). How *S. purpurea-root-acetone* can co-act with 5-FU to enhance the chemosensitivity of 5-FU is unclear and should be elucidated.

The American pitcher plant family *Sarraceniaceae* comprises three genera of pitcher plants with at least 35 species: *Darlingtonia*, *Heliamphora*, and *Sarracenia* [83]. We found the pharmacological potentials of *S. purpurea*. It needs to be explored as to whether other pitcher plant extracts also have these capacities.

In conclusion, we evaluated the TPC, TFC, cytotoxicity, and antioxidant activities of different parts of *S. purpurea* extracts by using methanol, ethanol, acetone, and distilled water. The cytotoxic effects of *S. purpurea-root-acetone* on the survival, apoptosis, proliferation, and migration of 4T1 cells were examined. We also determined the ingredients in *S. purpurea-root-acetone* by GC–MS. These results might indicate the pharmacological potentials of *S. purpurea* for further clinical anticancer chemotherapies.

## 4. Materials and Methods

### 4.1. Chemicals, Cell Lines, and Bacterial Strains

Cell culture medium, fetal bovine serum (FBS), and supplements were obtained from Gibco Invitrogen Corporation (Carlsbad, CA, USA). The *Escherichia coli* strain BL21(DE3) pLysS (Novagen, UK) was used for protein expression. The cell lines 4T1 carcinoma and B16F10 murine melanoma were obtained from Food Industry Research and Development Institute, Hsinchu, Taiwan [17,18]. Cancer cells were grown in Dulbecco’s Modified Eagle Medium containing 10% FBS, 100 units/mL penicillin, and 100 μg/mL streptomycin in a humidified 5% CO_2_ atmosphere at 37 °C. All other chemicals were purchased from Sigma-Aldrich (St. Louis, MO, USA) and were of analytical grade.

### 4.2. Plant Materials and Extract Preparations

Whole plants of *S. purpurea* were obtained in September 2019. The leaves, stems, and roots of *S. purpurea* were collected, dried, cut into small pieces, and pulverized into powder. One gram of plant powder was placed into a 250 mL conical flask. To the flask, 100 mL of 100% methanol, ethanol, acetone, or distilled water were added; the flask was shaken on an orbital shaker for 5 h.

### 4.3. Determination of TPC

The quantification of TPC was carried out using the modified Folin–Ciocalteu method [84]. The absorbance of the blue color developed was measured at 750 nm by using a UV/VIS spectrophotometer (Hitachi U 3300, Hitachi High-Technologies, Tokyo, Japan) [18]. The results were compared with the standard curves of gallic acid (GAE) and were expressed as mg equivalent/g dry weight.

### 4.4. Determination of TFC

The quantification of TFC was carried out using the aluminum chloride colorimetric method [85]. The absorbance of extracts and standard solutions was measured at 510 nm by using a UV/VIS spectrophotometer (Hitachi U 3300, Hitachi High-Technologies, Tokyo, Japan) [18]. The results are expressed as mg of quercetin (QUE) equivalent/g dry weight.

### 4.5. Determination of Antioxidant Activity by DPPH Radical Scavenging Assay

The plant extracts were assessed for their antioxidant activities using a DPPH assay [86]. DPPH free radical scavenging activity was determined using the formula: %Radical scavenging activity = (Control OD − Sample OD)/Control OD × 100. The absorbance was measured at 517 nm.

### 4.6. GC–MS Analysis

To determine the molecular composition of the sample, a GC–MS analysis was performed. The filtered sample was analyzed using Thermo Scientific TRACE 1300 Gas Chromatograph with a Thermo Scientific ISQ Single Quadrupole Mass Spectrometer system. The column employed in this experiment was Rxi-5ms (30 m × 0.25 mm i.d. × 0.25 μm film). Helium was used as the carrier gas at a constant flow rate of 1 mL/min. The initial oven temperature was 40 °C and it was maintained at this temperature for 3 min; the temperature was gradually increased to 300 °C at a rate of 10 °C/min and was maintained for 1 min. The temperature of the injection port was 300 °C. The compounds discharged from the column were detected by a quadrupole mass detector. The ions were generated by the electron ionization method. The temperatures of the MS quadrupole and source were 150 and 300 °C, respectively, electron energy was 70 eV, the temperature of the detector was 300 °C, the emission current multiplier voltage was 1624 V, the interface temperature was 300 °C, and the mass range was from 29 to 650 amu. The relative mass fraction of each chemical component was determined by the peak area normalization method. Compounds were identified by matching generated spectra with NIST 2011 and Wiley 10th edition mass spectral libraries.

### 4.7. Trypan Blue Cytotoxicity Assay

The trypan blue cytotoxicity assay was performed to assess cell death [87]. The 4T1 and B16F10 cells were seeded in 96-well plates at a density of 1 × 10^4^ and incubated with different extracts in 100 μL of volume [17,18]. After 24 h, the anticancer potentiality exhibited by the extract was estimated by performing a trypan blue cytotoxicity assay.

### 4.8. Chromatin Condensation Assay

The apoptosis in 4T1 and B16F10 cells was assayed with Hoechst 33342 staining [88]. The cells were seeded in 6-well plates at a density of 5 × 10^5^ cells per well in a volume of 100 mL of culture medium. Cells were allowed to adhere for 16 h. After different treatments, cells were incubated for an additional 24 h, washed with PBS, stained with the Hoechst dye (1 μg/mL) in the dark at RT for 10 min, and imaged using an inverted fluorescence microscope (Axiovert 200 M; Zeiss Axioplam, Oberkochen, Germany) at excitation and emission wavelengths (λ_em_) of 360 and 460 nm, respectively [17,18]. The apoptotic index was calculated as follows: apoptotic index = apoptotic cell number/(apoptotic cell number + nonapoptotic cell number).

### 4.9. Clonogenic Formation Assay

A clonogenic formation assay [17,89] was used to assess the 4T1 cell growth to study whether *S. purpurea-root-acetone* could inhibit 4T1 cell proliferation. Briefly, 4T1 cells were seeded on 6-well plates at a density of 1 × 10^3^ cells per well. After different treatments, plates were incubated for 5–7 days to allow clonogenic growth. After washing with PBS, colonies were fixed with methanol and stained with 0.5% crystal violet for 20 min, and the number of colonies was counted under a light microscope.

### 4.10. Wound-Healing Assay

An in vitro migration (wound healing) assay [17,90] was performed to study whether *S. purpurea-root-acetone* could inhibit 4T1 cell migration. Briefly, 4T1 cells were seeded in 24-well plates, incubated in serum-reduced medium for 6 h, wounded in a line across the well with a 200 μL pipette tip, and washed twice with the serum-reduced medium. After different treatments, cells were incubated for 24 h to allow migration.

### 4.11. Antibacterial Activities

The antibacterial activities of different extracts from *S. purpurea* were analyzed using an agar well diffusion assay [18,91]. Colonies of bacteria (*S. aureus*, *P. aeruginosa*, and *E. coli*) were diluted to prepare a 0.1 McFarland standard suspension. Then, the bacteria were inoculated into sterile Petri dishes of 60 mL of Mueller–Hinton agar plates. The plates were shaken gently to allow even mixing of bacterial cells and agar. All samples were dissolved in 30% DMSO to furnish 22 mg/mL. Exactly 90 μL of each extracted sample (6.0 mm diameter disc) was transferred onto the plate and incubated at 37 °C for 12 h. The diameters of the inhibition zones were recorded. The inhibition zone is an indication of the antibacterial activity, which increases in size as the potency of the extract increases.

### 4.12. Protein Expression and Purification

The construction of the huDHOase expression plasmid was reported [17,64]. The recombinant protein was purified using the protocol described previously for huDHOase [17,64]. Briefly, *E. coli* BL21(DE3) cells were transformed with the expression vector, and the overexpression of the expression plasmid was induced by incubating with 1 mM of isopropyl thiogalactopyranoside. The protein was purified from the soluble supernatant by using Ni^2+^-affinity chromatography (HiTrap HP; GE Healthcare Bio-Sciences), eluted with buffer A (20 mM Tris–HCl, 250 mM imidazole, and 0.5 M NaCl, pH 7.9), and dialyzed against a dialysis buffer (20 mM Tris–HCl and 0.1 M NaCl, pH 7.9). The protein purity remained at >97% as determined using SDS–PAGE (Mini-PROTEAN Tetra System; Bio-Rad, CA, USA).

### 4.13. Enzyme Assay

A rapid spectrophotometric assay was used to determine the activity of huDHOase [17,40,59,92,93,94]. Briefly, the hydrolysis of DHO was measured at 25 °C, and the decrease in absorbance at 230 nm. The purified huDHOase was added to a 2 mL solution containing 0.2 mM DHO and 100 mM Tris–HCl at pH 8.0 to start the reaction. The hydrolysis of DHO was monitored using a UV/Vis spectrophotometer (Hitachi U 3300; Hitachi High-Technologies, Tokyo, Japan).

### 4.14. Molecular Docking Studies

7,8-Dihydro-α-ionone, 24-norursa-3,12-diene, α-amyrin, stigmast-5-en-3-ol, betulin, and 24-noroleana-3,12-diene were docked for their binding capacities in huDHOase via MOE-Dock [46]. Before starting the docking process, the water molecules present in the crystal structure of huDHOase (PDB ID 4C6C) were removed via MOE. Hydrogen atoms were added to the protein structure through 3D protonation with subsequent minimization of energy. Top-ranked confirmations were developed and analyzed.

## Figures and Tables

**Table 1:** TPC of *S. purpurea* extracts.

	Leaves	Stems	Roots
Water	5.0 ± 0.3	6.0 ± 0.7	3.2 ± 0.2
Methanol	14.4 ± 0.6	15.0 ± 0.7	10.2 ± 0.3
Ethanol	12.5 ± 0.5	14.7 ± 0.9	12.1 ± 0.3
Acetone	14.8 ± 0.4	20.5 ± 0.8	33.4 ± 0.7

**Table 2:** TFC of *S. purpurea* extracts.

	Leaves	Stems	Roots
Water	10.8 ± 0.6	12.5 ± 0.4	5.6 ± 0.4
Methanol	36.7 ± 2.0	60.8 ± 1.6	58.0 ± 1.1
Ethanol	39.1 ± 2.3	61.0 ± 1.8	62.0 ± 1.5
Acetone	39.7 ± 1.6	66.9 ± 1.7	107.9 ± 2.2

**Table 3:** Antioxidant activities of *S. purpurea* extracts.

	IC_50_ (μg/mL)		
Solvent	Leaves	Stems	Roots
Methanol	289.4 ± 4.0	251.2 ± 3.0	126.0 ± 1.2
Ethanol	328.2 ± 3.8	245.2 ± 1.8	117.4 ± 2.6
Acetone	365.3 ± 4.2	233.4 ± 2.1	89.3 ± 2.2

IC_50_ values were calculated from the titration curves of the DPPH assay by determining the concentration of the extract needed to achieve the midpoint value for inhibition. Due to < 30% inhibition at a concentration of 600 μg/mL, we did not determine the IC_50_ values of the water extracts of *S. purpurea*.

**Table 4:** Inhibition zone of *S. purpurea* extracts.

		Zone of Inhibition (mm)		
Material	Solvent	*E. coli*	*P. aeruginosa*	*S. aureus*
Leaves	Water	0	0	0
	Methanol	11	9	0
	Ethanol	13	11	0
	Acetone	14	12	0
Stems	Water	0	0	0
	Methanol	11	9	7
	Ethanol	11	10	7
	Acetone	14	13	8
Roots	Water	0	0	0
	Methanol	11	9	7
	Ethanol	12	11	8
	Acetone	15	14	11

The water extracts of *S. purpurea* did not inhibit bacterial growth.

**Table 5:** Compounds detected by the GC–MS analysis of *S. purpurea-root-acetone*.

Peak No.	RT (min)	Name of Compounds	MF	CS	MW	Area (%)
1	16.55	7,8-Dihydro-α-ionone	C_13_H_22_O	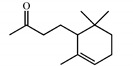	194	84.43
2	29.18	24-Norursa-3,12-diene	C_29_H_46_	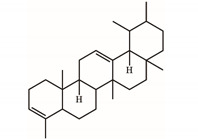	394	5.01
3	33.9	α-Amyrin	C_30_H_50_O	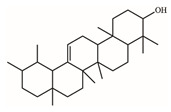	426	3.57
4	32.35	Stigmast-5-en-3-ol	C_29_H_50_O	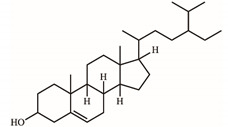	414	3.12
5	33.19	Betulin	C_30_H_50_O_2_	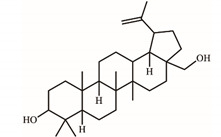	442	2.42
6	30.02	24-Noroleana-3,12-diene	C_29_H_46_	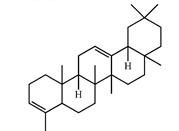	394	1.41

**Table 6:** Results of the docking studies against huDHOase.

Compounds	S Score	Receptor Residue	Interaction	Distance (Å)	E (kcal/mol)
Stigmast-5-en-3-ol	−6.8351	Asp1686	H-donor	2.77	−0.8
		Zn-α	Metal	2.07	−1.7
		Zn-β	Metal	2.25	−2.0
7,8-Dihydro-α-ionone	−6.3427	Zn-α	Metal	2.11	−1.8
		Zn-β	Metal	2.13	−1.7
α-Amyrin	−6.0751	Asn 1505	H-donor	2.97	−1.0
24-Noroleana-3,12-diene	−5.8697	His 1473	H-pi	4.42	−0.5
Betulin	−5.8655	Glu 1561	H-donor	3.34	−0.5
24-Norursa-3,12-diene	−5.6540	No important residue			

## Data Availability

Not applicable.
